# White light polarization sensitive optical coherence tomography for sub-micron axial resolution and spectroscopic contrast in the murine retina

**DOI:** 10.1364/BOE.9.002115

**Published:** 2018-04-05

**Authors:** Danielle J. Harper, Marco Augustin, Antonia Lichtenegger, Pablo Eugui, Carlos Reyes, Martin Glösmann, Christoph K. Hitzenberger, Bernhard Baumann

**Affiliations:** 1 Center for Medical Physics and Biomedical Engineering, Medical University of Vienna, Waehringer Guertel 18-20/4L, 1090 Vienna, Austria; 2 University of Veterinary Medicine Vienna, Core Facility for Research and Technology, Veterinaerplatz 1, 1210 Vienna, Austria

**Keywords:** (110.4500) Optical coherence tomography, (130.5440) Polarization-selective devices, (170.5755) Retina scanning, (170.0110) Imaging systems

## Abstract

A white light polarization sensitive optical coherence tomography system has been developed, using a supercontinuum laser as the light source. By detecting backscattered light from 400 – 700 nm, an axial resolution of 1.0 µm in air was achieved. The system consists of a free-space interferometer and two homemade spectrometers that detect orthogonal polarization states. Following system specifications, images of a healthy murine retina as acquired by this non-contact system are presented, showing high resolution reflectivity images as well as spectroscopic and polarization sensitive contrast. Additional images of the very-low-density-lipoprotein-receptor (VLDLR) knockout mouse model were acquired. The high resolution allows the detection of small lesions in the retina.

## 1. Introduction

The axial resolution of an optical coherence tomography (OCT) system is directly proportional to the square of the central wavelength of the light source, and inversely proportional to the full width at half maximum (FWHM) of the source’s spectral bandwidth [1]. The development of supercontinuum lasers has therefore opened up a fresh wave of spectral domain (SD-) OCT. Higher repetition rates and lower relative intensity noise fluctuations have now made it possible to perform OCT with broader spectral bandwidths [2, 3] and lower central wavelengths [4–7], thus resulting in a better axial resolution.

OCT has proven itself time and time again as being an important technique in retinal imaging, both in the clinic in human subjects [8] and in preclinical imaging of healthy animals as well as in models of diseases [9]. Despite the lack of a fovea, the murine retina shows many similarities to that of a human retina [10], particularly in its layer structure [11]. The mouse is therefore often used as an animal model, as they are cheap and easy to look after and can be bred quickly. Their short lifespan means that if their genome is manipulated, the pathologies associated with this progress more quickly than they would in other animals with longer lifespans, thus resulting in shorter experiment times if a longitudinal study of the disease progression is to be performed. To be able to characterize the progression of a disease (in humans or animal models) with imaging techniques, it is important that the resolution is as high as it possibly can be in order to detect more subtle changes.

However it is not only higher resolution which plays a role in better image interpretation. In conventional OCT reflectivity images of the retina, it can often be difficult to distinguish pathological features if their particle distribution and backscattering properties are similar to that of the surrounding tissue. In this case, functional extensions of OCT are often applied to highlight contrasts which are not visible in the reflectivity images. Doppler OCT [12, 13], for example, is often used to quantify blood flow in the retina by performing differential phase analysis between successive A-scans (or B-scans). This method detects motion along the axis of the probing beam, resulting in contrast between the moving red blood cells and their static surrounding tissue [14–16]. OCT angiography (OCTA) can also be used to visualize blood flow, but this time by looking at either phase or intensity variations to detect decorrelation [17, 18]. In the retina, this results in a detailed map of where the blood flow occurs down to the capillary level [19].

To further quantify blood vessels in terms of blood oxygen saturation, a spectroscopic analysis can be performed on visible light OCT data, visualizing the behavior of different wavelengths corresponding to the absorption peaks of oxyhemoglobin and deoxyhemoglobin [20–24]. While this can also be done at infrared wavelengths, the effect is much more pronounced in the visible light range [25], and since the central wavelengths are lower a better axial resolution is maintained [7]. Although these studies have been performed in blood vessels, the method is not dependent upon the movement of blood cells and can be performed in other tissues. If the wavelength range is extended to the full visible light spectrum, it is also possible to reconstruct “true color” images if the wavelengths chosen fall at red, green and blue, providing a different contrast in static tissue capable of chromophore detection [26]. It has already been shown that scattering properties of retinal layers can change with wavelength [27] and therefore visible light OCT could play a role in a spectroscopic investigation of these layers.

Also enhancing the contrast in static tissue, polarization sensitive OCT (PS-OCT) [28, 29] highlights the intrinsic contrast caused by the fact that some tissues change the polarization state of light incident upon them. In the healthy retina of both humans and mice, for example, the retinal pigment epithelium (RPE) depolarizes light due to the melanin granules which are present in this layer [30, 31]. Each granule scatters the light and alters the polarization state, and the cumulative effect of the interaction of light with many granules is a random polarization state, given that the pigment density is sufficient. This is known as depolarization [32, 33]. It therefore follows that if there are any subtle irregularities in the shape of the RPE, this can also be detected by PS-OCT.

Previous studies have shown the use of PS-OCT in human retinal imaging for both healthy volunteers [34–39], and in patients with diseases such as age-related macular degeneration (AMD) [40–45], glaucoma [35, 39, 46] and choroidal nevus [42]. Recent PS-OCT studies in our group have focused mainly on rodent models of such diseases. Since intraocular pressure is an important parameter in glaucoma [47], this was studied in the rat eye using a high resolution PS-OCT system (axial resolution = 3.8 µm in retinal tissue) described in [48]. It was shown that scleral birefringence, a property defined by the axis orientation of collagen fibers, could be correlated to the intraocular pressure [49]. The system used to perform this study was further used to image the very-low-density-lipoprotein-receptor (VLDLR) mouse model which forms type 3 neovascularizations similar to those found in patients with retinal angiomatous proliferation, a form of neovascular AMD [50]. In this case, the melanin displacement was visualized with PS-OCT over time in a longitudinal study [51].

In this work, we present a new PS-OCT system operating across the whole visible light range with an axial resolution which is approximately five times higher than in our previous system. As the system was specifically designed for mouse retinal imaging, we discuss its design, measure its specifications and finally we demonstrate reflectivity, phase retardation and spectroscopic retinal images of both healthy mice and the VLDLR knockout mouse model as acquired by the system.

## 2. Materials and methods

### 2.1. System

**Fig. 1:**
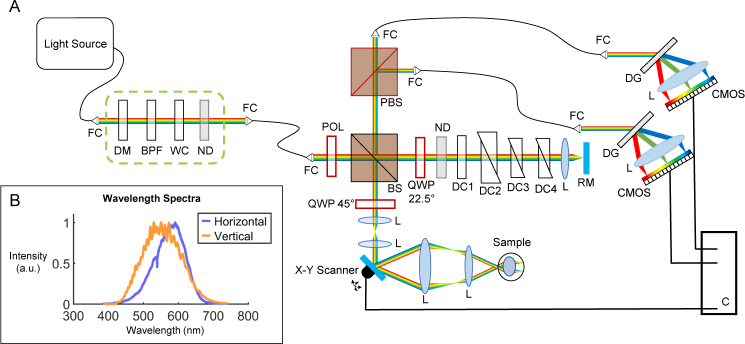
(a) Diagram of the white light PS-OCT system. Polarization optics are drawn in red. **FC** Fiber Collimator. **DM** Dichroic Mirror. **BPF** Bandpass Filter. **WC** Water Cuvette. **ND** Neutral Density Filter. **POL** Polarizer. **BS** Beam Splitter. **QWP** Quarter Wave Plate. **DC1** Dispersion Compensation Water Cuvette. **DC2–4** Dispersion Compensation Prisms. **RM** Reference Mirror. **L** Lens. **PBS** Polarizing Beam Splitter. **DG** Diffraction Grating. **CMOS** Line Scan Camera. **C** Computer. (b) Wavelength range detected by the two spectrometers (reference spectra).

A white light PS-OCT system was developed as shown in Fig. 1(a)

. A supercontinuum laser (EXU6, NKT Photonics) was chosen as the light source, with the desired spectrum (400 – 700 nm) selected using a homemade filter box (indicated in the green dashed rectangle) consisting of a dichroic mirror (DMSP805, Thorlabs), a bandpass filter (FESH0750, Thorlabs), a water cuvette (lightpath = 10 mm) and a neutral density (ND) filter (optical density = 1.8). As the dichroic mirror and the bandpass filter break down at longer wavelengths (>1500 nm), the water cuvette was added to absorb the remaining infrared part of the spectrum. Heat sinks were attached to the water cuvette for efficient heat dissipation. The neutral density filter was then added to attenuate the remaining beam power in the visible light range before passing the beam through a photonic crystal fiber (SuperK FD7, NKT Photonics) to the interferometer. The photonic crystal fiber was necessary to maintain single mode transmission over the whole wavelength range.

The incident white light beam was first linearly polarized by a Glan-Thompson polarizer (5524, Newport Spectra-Physics) and then separated into a sample and reference arm by a 50/50 beam splitter. In the sample arm, a variable telescope (AC127-030-A, focal length = 30 mm and AC127-025-A, focal length = 25 mm, Thorlabs) was used to correct for poor focus of each individual mouse eye during the measurement. A set of X-Y galvanometric scanners (GVS002, Thorlabs) were then used in combination with a final telescope (AC508-075-A, focal length = 75 mm and AC254-030-A, focal length = 30 mm, Thorlabs) to scan a beam with a 1/*e*^2^ diameter of 0.3 mm through the anterior eye. The system then relied on the natural optics of the mouse eye to focus the light onto the retina. Such a setup does not require any physical contact with the mouse eye. In the reference arm, glass prisms of various glass types (similar to those in the lenses in the sample arm) were added to compensate for the phase dispersion introduced by the two telescopes. A water-filled cuvette (type 96 X-rite colorimeter cell, lightpath = 2.5 mm, FireflySci) was also added to loosely compensate for the dispersion introduced by the eye itself.

Polarization optics were also added to the interferometer. In the sample arm, the beam passed through a quarter wave plate (QWP) (10RP44-1, Newport Spectra-Physics) angled at 45° to illuminate the sample with circularly polarized light. A change in polarization state caused by the sample generally results in an elliptical state returning to the beam splitter, which interferes with the beam returning from the reference arm (which itself has traversed a QWP at 22.5° twice). A polarizing beam splitter then splits the resultant beam by polarization state, allowing a polarization sensitive detection. Each beam passes through a photonic crystal fiber (SuperK FD7, NKT Photonics) to a homemade spectrometer, comprising a diffraction grating (1800 lines/mm), a custom designed lens and a CMOS line scan camera (ELiiXA+ 16k, e2v) with four rows of 16384 pixels, each measuring 5 µm × 5 µm. However as the diameter of the diffraction limited spot size of the beam lies between 6 – 9 µm (depending on the wavelength), the pixels were binned to 8192 pixels of 10 µm × 10 µm, resulting in a spectrometer resolution of 0.044 nm/pixel. The wavelength spectrum as acquired by the spectrometers is shown in Fig. 1(b).

Data from the CMOS line scan cameras were sent to the computer via a frame grabber (Komodo, Kaya Instruments) over a CoaXPress [52] link. The acquisition and the X-Y galvanometric scanners were synchronized and controlled by LabVIEW (Version 15.0f2, 64-bit, National Instruments). For 3D data acquisition, the scanners were set to execute a smoothed raster scan pattern with 512 × 400 data points at an A-scan rate of 25 kHz. Although the maximum line rate of the cameras was 40 kHz (for acquisition with 12 bit resolution), the A-scan frequency was sacrificed in favor of the exposure time and therefore the signal-to-noise ratio (SNR).

### 2.2. Data acquisition and post processing

#### 2.2.1. Reflectivity: backscattering based imaging

Following acquisition, each data set was processed using a combination of MATLAB (R2015b, MathWorks) and Fiji (ImageJ 1.51p) [53]. To obtain standard reflectivity OCT images based on backscattering, the background is first removed by subtracting the average spectrum of the whole B-scan and then the spectral data is resampled to be linear in k-space. Prior to processing of the second channel, the data is first passed through an equation of the form *y* = *mx* + *c* in order to align the spectrum to that of the first channel (previously calibrated using color filters), and a normalization is performed to correct for the intensity differences between channels at each wavelength. Numerical dispersion compensation is then applied using a variation on the method described by Wojtkowski et al. [54] (see section 2.2.2), and the Fourier transform is computed. In some cases, multiple frames are averaged for speckle reduction. The reflectivity images are then calculated by summing the squares of the signals from each channel [55].

#### 2.2.2. Dispersion compensation for broad bandwidth

A method for dispersion compensation involving the addition of a phase correction term to the complex analytic representation of the spectral fringe pattern has previously been described [54]. In this method, the coefficients *a*_2_ and *a*_3_ are adjusted to balance the second- and third-order dispersion terms, respectively. However, due to the broad spectral bandwidth in the white light OCT system, the numerical constants *a*_2_ and *a*_3_ are themselves wavelength dependent and can differ by up to an order of magnitude across the whole wavelength range. The correction terms were therefore calculated for three separate wavelength ranges, (*λ*_1_, *λ*_2_, *λ*_3_), independently, and the phase correction curves for these wavelengths were then concatenated and smoothed to ensure no discontinuities. In doing this, a first order dispersion term is introduced which must again be corrected for. This technique is a generalization of that shown in Eq. 1, where the phase correction term in k-space, $\bar{\Phi }$, is dependent not only on the central wavenumber *k*_0_ and the wavenumber *k*, but also on wavelength dependent constants. The generalization of this case to three wavelength ranges means that the exact relationship between the numerical constants and the wavelength range need not be known and a trial and error approach can be taken for each data set, finding the phase correction constants by judging the quality of the images by eye. In this case, the *a*_1_ term is also required to ensure the image appears at the same position in depth for each wavelength range, correcting for first order dispersion.

$$\bar{\Phi }(k,\lambda_{i},i=1,2,3)=a_{1}(\lambda_{i})\times (k-k_{0})-a_{2}(\lambda_{i})\times {\left(k-k_{0}\right)}^{2}-a_{3}(\lambda_{i})\times {\left(k-k_{0}\right)}^{3}$$ (1)

As this process is rather lengthy, it was not performed for all data sets. For the remainder, dispersion compensation was performed exactly as outlined in [54].

#### 2.2.3. Polarization sensitive image processing

After all spectrum alignment and normalization (see section 2.2.1), the phase retardation for each pixel, *δ*, can be calculated [28, 55]:

$$\delta =\arctan\left(\frac{A_{V}}{A_{H}}\right)$$ (2)

In this equation, *A_V_* and *A_H_* are the amplitudes of the vertically and horizontally polarized channels, respectively. Prior to calculating the retardation, an intensity threshold was set in the corresponding reflectivity image (≈ 3 – 5 dB above the mean noise level), below which the retardation was not calculated. This ensured that the retardation image consisted only of retinal signal, removing the background noise.

#### 2.2.4. Spectroscopic OCT

The original spectra were filtered by Gaussian windows centered at blue (460 nm), green (550 nm) and red (640 nm) wavelengths to gain an additional spectroscopic contrast. The filtering was performed after resampling of the spectra to k-space to ensure a constant axial resolution (≈ 4 µm in tissue) for each wavelength channel. The resulting images were first normalized by histogram shape, and then combined to form an RGB image - a “true color” representation of the OCT data [26]. As different wavelengths produce different speckle patterns in the OCT images, each wavelength channel B-scan was smoothed by a mean filter (rectangular kernel, 3 × 2) before the RGB image was created.

### 2.3. Mice

Both healthy adult control mice (from a BL/6 background) and very-low-density-lipoprotein-receptor (VLDLR) knockout mice (8 – 10 months old) were measured using the system. The mice were anesthetized using an isoflurane/oxygen mixture and pupils were dilated using tropicamide and phenylephrine, topically administered mydriatic agents. The eyes were kept moist during the experiments using artificial tear drops. A stage was designed and built to position the mouse and to hold the anesthesia apparatus to the nose for the duration of the experiments. All experiments were performed in accordance with the Association for Research in Vision and Ophthalmology (ARVO) Statement for the Use of Animals in Ophthalmic and Vision Research, and under a protocol approved by the ethics committee of the Medical University of Vienna and the Austrian Federal Ministry of Science, Research and Economy (BMWFM-66.009/0360-WF/V/3b/2016).

## 3. Results

### 3.1. System characterization

#### 3.1.1. Specifications

**Fig. 2:**
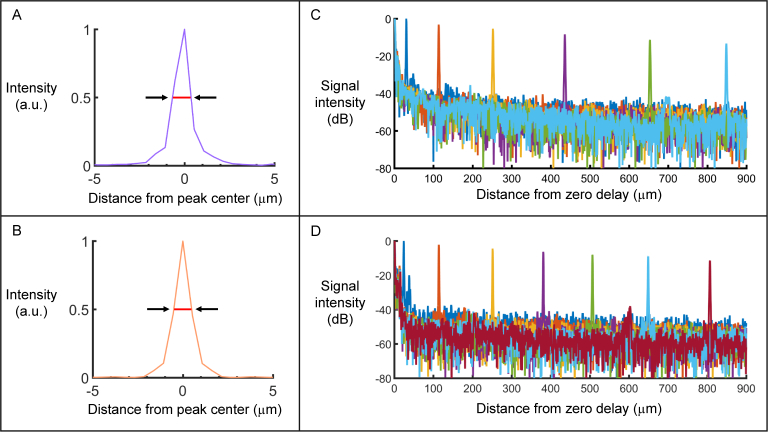
(a – b) Axial resolution measurement in (a) the horizontally polarized channel and (b) the vertically polarized channel. Red lines indicate the full width at half maximum (FWHM) of the signal intensity which is equal to 1.0 µm in air for both channels. (c – d) Sensitivity roll-off as a function of depth (in air) for (c) the horizontally polarized channel (roll-off decay ≈ 14 dB/mm) and (d) the vertically polarized channel (roll-off decay ≈ 15 dB/mm).

The axial resolution of the system was measured by placing a mirror in the sample arm and measuring the FWHM of the resultant point spread function after Fourier transformation. Spectral phase correction as described in detail by Choi et al. [56] was performed to remove dispersive effects. An axial resolution of 1.0 µm in air was measured in both channels, which corresponds to 0.73 µm in rodent retinal tissue where the refractive index is assumed to be 1.37 [57]. Figure 2(a)

shows the axial resolution in the horizontally polarized channel, while Fig. 2(b) displays the same in the vertically polarized channel.

The sensitivity and corresponding roll-off of the system were also measured. This was done by placing a ND filter of known optical density in the sample arm in front of a mirror, and calculating the maximum SNR which could be achieved at different depth positions. A maximum sensitivity of 96 dB in both channels was calculated (with an incident power of 1 mW) by adding the SNR to the attenuation factor introduced by the ND filter. Figure 2(c – d) shows the result of the sensitivity roll-off as a function of depth for both the horizontally (c) and vertically (d) polarized channels. The corresponding sensitivity roll-off was 14 dB/mm in the horizontally polarized channel and 15 dB/mm in the vertically polarized channel. The total imaging depth range was measured to be 2.2 mm, corresponding to ≈ 1.6 mm in retinal tissue. However since the thickness of the murine retina is < 300 µm, only the first 700 µm of the depth range was used for imaging.

#### 3.1.2. Polarization measurement validation

**Fig. 3:**
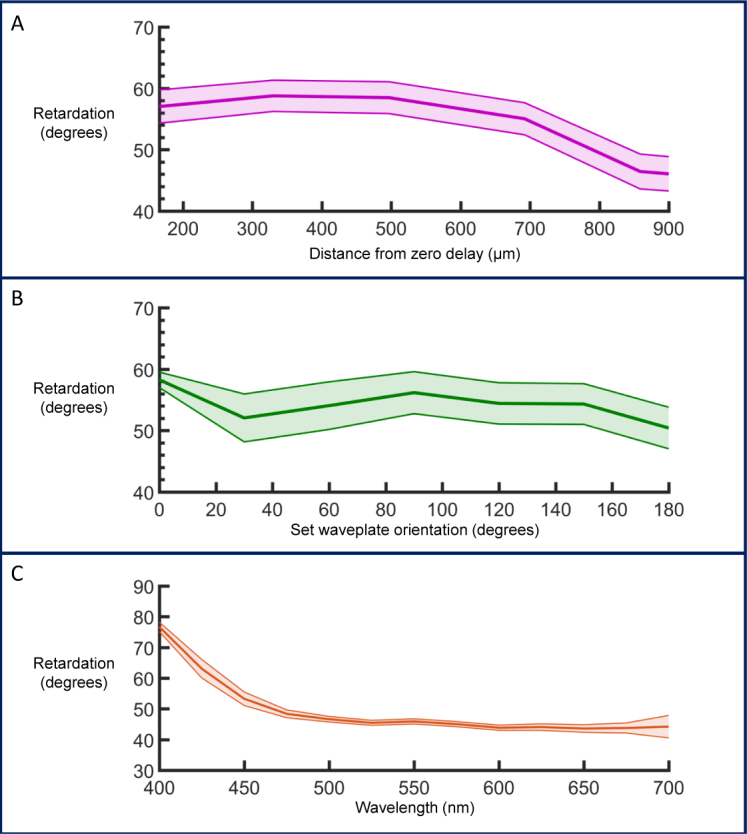
(a) Phase retardation at different depth positions. (b) Phase retardation at different waveplate orientations. The retardation stays within ± 4° for a 180° rotation of the waveplate. (c) A wavelength dependence on retardation is observed with a smaller phase retardation at longer wavelengths. The error associated with the measurement is greater at the edges of the spectrum. All graphs display mean retardation ± propagated standard deviation.

In order to verify the capability of the system to resolve polarization properties of a sample, a phantom was constructed consisting of a QWP designed for 1300 nm and a mirror, as described in [55]. The retardation of this phantom was then measured at different distances from the zero delay and also for different orientations of the QWP. Figure 3(a)

shows the retardation values as a function of distance from the zero delay. Within the depth range used for murine retinal imaging (the first 700 µm) the measured retardation value stays approximately constant and does not go outwith ± 2°. For the retardation as a function of set waveplate orientation (Fig. 3(b)), the value is also relatively constant. The slight periodic shift (± 4°) is most likely due to a misalignment of the waveplate with respect to the beam. This would also explain the discrepancy between the measurements at 0° and 180°, which should theoretically be identical.

The acquired spectra were then filtered by Gaussian windows to sample the phase retardation at different central wavelengths ranging from 400 – 700 nm. The retardation shows a strong wavelength dependence, particularly in the region of 400 – 500 nm. The graph in Fig. 3(c) demonstrates the measurements for one particular depth position and waveplate axis orientation, but the trend is reproducible at different depths and orientations.

In this section all measurements were repeated 512 times without scanning the mirror, and the mean and standard deviation of the intensity values of each channel were calculated. The standard deviation of each channel was propagated through the retardation equation (Eq. 2) [58], and the mean retardation at each point is plotted with error bars indicating the associated error values.

### 3.2. Imaging the healthy murine retina

**Fig. 4:**
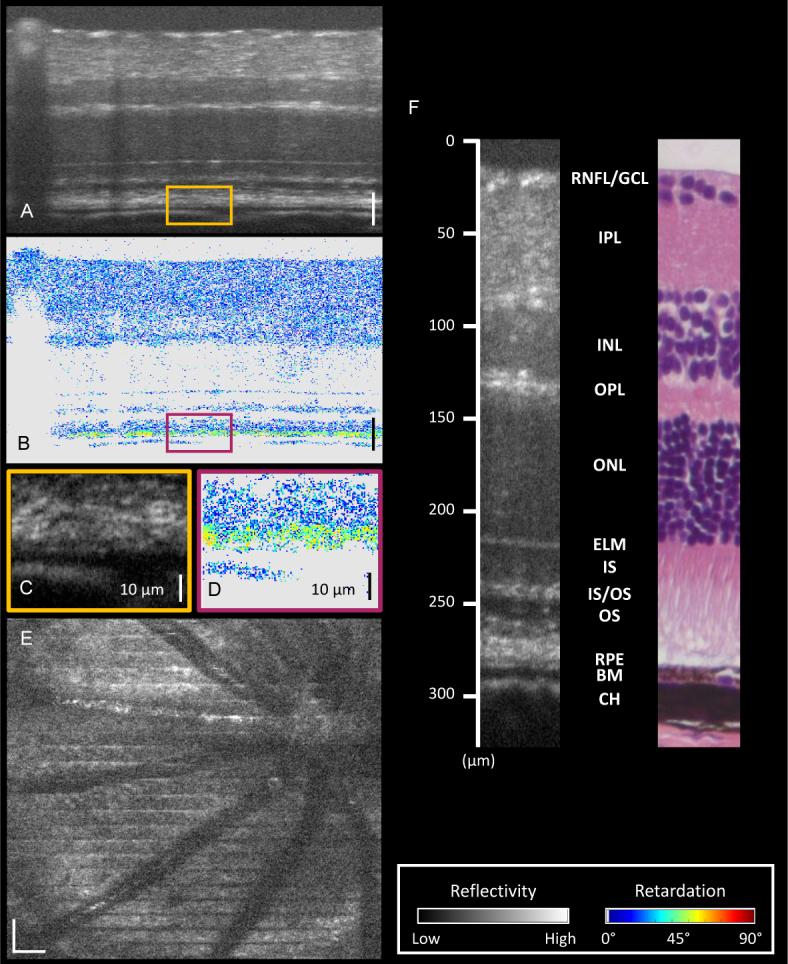
(a) Reflectivity image acquired by the white light PS-OCT system. (b) Corresponding phase retardation image. Most of the healthy murine retina is polarization preserving. (c) Zoomed in region indicated by the orange box in (a). The depolarizing RPE is difficult to distinguish from its surrounding layers. (d) Zoomed in region indicated by the purple box in (b). High resolution PS-OCT highlights the depolarizing RPE. (e) An en-face projection of a region of a healthy mouse retina including the optic nerve head (ONH). (f) A section of a mouse retina as measured with white light OCT, and a histology image of a similar area from a different mouse. Depth positions are marked and retinal layers are labeled. **RNFL** Retinal nerve fiber layer. **GCL** Ganglion cell layer. **IPL** Inner plexiform layer. **INL** Inner nuclear layer. **OPL** Outer plexiform layer. **ONL** Outer nuclear layer. **ELM** External limiting membrane **IS** Inner segments. **IS/OS** Inner segment/outer segment junction. **OS** Outer segments. **RPE** Retinal pigment epithelium. **BM** Bruch’s membrane. **CH** Choroid. All scale bars correspond to 50 µm unless otherwise stated. Color maps for reflectivity (grayscale) and retardation (colored) can be found on the bottom right. Pixels which appear gray in the retardation images indicate that the retardation value was not calculated as the SNR was not high enough.

The white light PS-OCT system was used to image the retina of healthy adult mice. An example of a 30× averaged reflectivity B-scan image can be found in Fig. 4(a)

. The corresponding phase retardation image is seen in Fig. 4(b). Most of the mouse retina is polarization-preserving and therefore appears blue in the image. However when zooming in to the region including the RPE (reflectivity: Fig. 4(c), retardation: Fig. 4(d)), the addition of PS contrast immediately highlights the boundary of the depolarizing RPE, which is difficult to distinguish from the surrounding layers in the standard reflectivity images. An example of an en-face projection over the whole depth of the retina, including the optic nerve head, can be found in Fig. 4(e). The adjustable focusing telescope in the sample arm allows a tighter beam focus on the retina and therefore the vessels appear well-defined in the image. The horizontal stripes in the image are caused by breathing artifacts. In order to highlight the axial resolution, Fig. 4(f) shows a labeled section of the retina as measured with white light OCT plotted against depth position, and a histological sample of a similar region of the healthy mouse retina. The high resolution allows for clear retinal layer definition. Since the external limiting membrane (ELM) is a reflective boundary between two surfaces, its apparent thickness in OCT images can be considered as a measured in vivo axial resolution. In single frames, this corresponds to 0.91 – 0.97 µm. The discrepancy between this value and the theoretical value of 0.73 µm can be explained by imperfect dispersion compensation and attenuation of the edges of the wavelength spectrum with depth in the retina.

### 3.3. Spectroscopic OCT

**Fig. 5:**
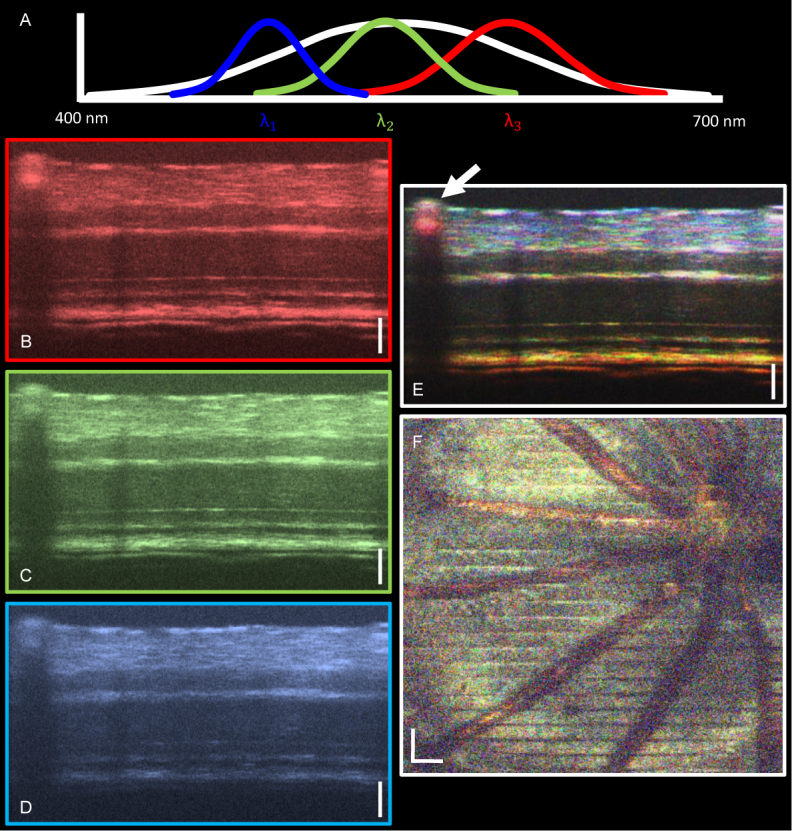
(a) A cartoon representation of spectral filtering in wavelength space. The original spectrum is multiplied by three Gaussian windows centered at blue (*λ*_1_ = 460 nm), green (*λ*_2_ = 550 nm) and red (*λ*_3_ = 640 nm) wavelengths. (b–d) Red (b), green (c) and blue (d) B-scan images after spectral filtering. (e) “True color” RGB image produced by the normalized and smoothed addition of the red, green and blue channels. White arrow indicates blood vessel. (f) Spectroscopic en-face projection over the whole retina including the optic nerve head. Vessels are immediately highlighted by color contrast. All scale bars correspond to 50 µm.

Spectroscopic analysis, as represented in Fig. 5(a)

, was performed on the OCT images; sacrificing axial resolution to gain a spectroscopic contrast. The Gaussian windows were selected to give an axial resolution of 4 µm in retinal tissue at each wavelength. Figure 5(b–f) shows the results of such an analysis applied to the same image as in Fig. 4(a). The acquired spectra were filtered using Gaussian windows centered at red (Fig. 5(b)), green (Fig. 5(c)) and blue (Fig. 5(d)) wavelengths. From these images it is clear that the penetration depth is greatest in the red channel, and decreases with decreasing wavelength. By combining the three channels to form a “true color” RGB image (Fig. 5(e)), blood vessels are immediately highlighted as there is a greater component of backscattered light at red wavelengths compared to the others.

The spectroscopic analysis was also performed on 3D datasets and en-face projection maps were made across the whole depth of the retina (Fig. 5(f)). Again the vessels are immediately highlighted. This could prove a useful method for identifying blood without relying on the cellular motion to provide contrast.

### 3.4. VLDLR knockout mouse model

**Fig. 6:**
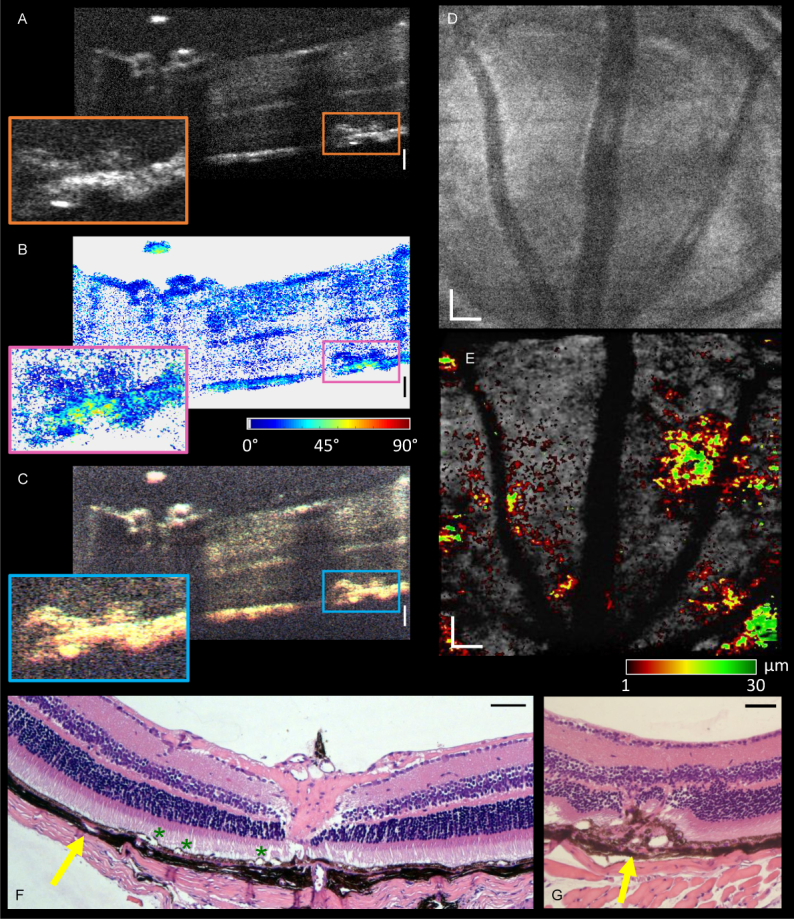
(a) Reflectivity B-scan of the VLDLR knockout mouse model as acquired by white light OCT (4× average of consecutive B-scans). The orange box indicates the presence of a retinal lesion. (b) The corresponding phase retardation image. The PS contrast indicates the position of the lesion, but in this case there is no indication of depolarizing melanin in the photoreceptor layer. (c) The true color spectroscopic RGB image. There appears to be some color contrast within the lesion. (d) En-face projection across an area of the retina superior to the optic nerve head. There is no indication of retinal lesions. (e) En-face projection from the top of the RPE (grayscale) with a heat map of height above the RPE layer where additional abnormal signal is seen. This highlights the lesion area. (f) Representative histology image of the retina of the VLDLR knockout mouse model. Yellow arrow indicates a miniscule and eosinophilic thickening between the RPE and choroid, which potentially features a lesion. High resolution imaging would be required to study the progression of this in vivo. Green asterisks indicate artefactual detachment. (g) A larger lesion (indicated by yellow arrow) which shows melanin has been displaced to the outer nuclear layer, and the whole layer structure is disrupted. All scale bars correspond to 50 µm. Insets in (a-c) show a depth of 100 µm.

The white light PS-OCT system was also used to image the retina of the VLDLR knockout mouse model which is known to develop retinal lesions [59]. The tomogram in Fig. 6(a)

shows the standard reflectivity image in which a lesion (inset) can be found. When zooming into the abnormal area (indicated by the orange box, 100 µm high), the lesion clearly has some structure which indicates RPE disruption. Figure 6(b) shows the phase retardation image of the B-scan. The inset shows that while the RPE is disrupted somewhat, the depolarizing melanin pigments have not yet been displaced into the photoreceptor layer. Figure 6(c) displays the true color spectroscopic representation of the lesion. It would appear that there is some color contrast within the lesion, but a more comprehensive analysis would be required to confirm the source of this.

While an en-face projection over the whole depth of the retina (Fig. 6(d)) does not highlight any immediate irregularities, manual segmentation of the RPE layer alone shows the location of lesion sections. Figure 6(e) shows an en-face projection of all signal from the top surface of the RPE and deeper retinal layers in grayscale, and a heat map of height above the RPE layer (also manually segmented) where additional high-intensity signal (indicating presence of a lesion) is observed. Due to the high axial resolution of the system, the lesions can be detected and monitored using white light OCT with a much more precise level of detail when compared to infrared light. For comparison, Fig. 6(f) shows an example of a histology image of the retina of the VLDLR knockout mouse model. The yellow arrow indicates a miniscule and eosinophilic thickening between the RPE and choroid, the growth of which could be studied in more detail in vivo with the white light system. While preparing the histology images, artefactual detachments can occur during paraffin embedding of the eye cup. Examples of this are highlighted by the green asterisks in Fig. 6(f). Figure 6(g) shows a very large lesion which causes disruption of all deeper retinal layers. Since these mice are used as a model of age-related macular degeneration, a more precise mapping of the growth of these lesions could help to gain a better understanding of the disease.

## 4. Discussion

As the OCT system presented in this work makes use of backscattered light from 400 – 700 nm, the high axial resolution allows a 3D reconstruction of the mouse retina to be visualized in great detail. The additional contrast channels of polarization sensitivity and spectroscopic information open up new possibilities to visualize properties of the mouse retina for this wavelength range. As the eye is designed for visible light, “true color” images can be reconstructed, which may give a more intuitive idea of how the retina behaves since the wavelength spectrum used for imaging overlaps the absorption spectra of the photoreceptors in the retina [60, 61].

However the extension of OCT to the whole visible light range does not come without its challenges. In order to achieve the highest possible axial resolution, the dispersion must be corrected for to a high degree of accuracy. In this work, both hardware and software methods are used, and there is still a small amount of dispersion present in the images. The *a*_2_ and *a*_3_ parameters in Eq. 1 change from mouse to mouse, and therefore fully-automated post-processing has not yet been implemented.

The broad bandwidth also poses new challenges in the acquisition of the polarization sensitive data. As the detectors of the CMOS cameras in the spectrometers are 8.2 cm long, it was not possible to align both of them truly identically. The result of this was that an additional calibration step was required before Fourier transformation, aligning the spectra of the second channel to those of the first. Also, the polarization optics do not perform ideally across the whole wavelength spectrum which could lead to a slight offset in the retardation measurements. However it is not only the polarization optics which are being used at their limits. Every lens present in the system was modeled using Zemax (OpticStudio 15.0, Zemax LLC) to find a combination with minimal chromatic aberrations, and the lenses for the spectrometers were custom made. To extend the bandwidth of PS-OCT any further would require optical components which are not yet available off-the-shelf.

White light OCT also has its limitations, such as the limited penetration depth. As the melanin in the RPE is highly absorbing in the visible light range, there is very little light penetration to the choroid. While this system was designed specifically for the mouse eye, there is also the question about the transfer of white light OCT to humans [4]. As the human eye is very sensitive to shorter wavelengths, care must be taken to keep the power as low as possible, which in turn reduces the power incident upon the sample and therefore lowers the system sensitivity [4].

When performing the spectroscopic analysis, it is clear that the penetration depth decreases with decreasing wavelength and therefore the pure addition of the three channels results in a color gradient from white at the anterior surface of the retina to only red in the choroid (Fig. 5(e)). In order to correct for the effects of the spectral attenuation and the spectral sensitivity roll-off in the images, it would be possible to calculate the sensitivity roll-off at each wavelength and use this as a correction factor. However as the blue light signal intensity is low at the level of the RPE, this method significantly amplifies the noise and it is no longer possible to be confident of the spectroscopic data. For this reason, the three color channels were simply combined, with the idea that the spectroscopic contrast could be local to the surrounding tissue rather than global to the whole image. Although the signal is low in the blue channel in the posterior retinal layers, the anterior retinal layers allow a true color image to be reconstructed. The spectroscopic analysis indicates that there do appear to be structures in the RNFL/GCL which scatter only at specific wavelengths, but this needs further investigation. What is immediately clear is the vessel contrast due to the hemoglobin present in blood. If it is possible to detect blood in this manner, this could provide an interesting alternative to OCT angiography. In addition to highlighting the vessels, the spectroscopic analysis could also be used for leakage detection in the retina, or also in ex-vivo work, as the flow of blood is not necessary for contrast.

In addition to blood detection, the spectroscopic approach could also be used to detect other chromophores, such as melanin. As melanin pigment is brown, it would be reasonable to assume that it could also be identified by spectroscopic analysis, leading to a brownish appearance of the RPE and the remnant of the hyaloid canal which are both known to contain melanin [31, 62]. While this brownish hue can be somewhat seen in Fig. 5(e) (RPE) and (f) (hyaloid canal, top of ONH region), more analysis must be performed to confirm that melanin is the source of this. This could perhaps be explored by measuring the melanin concentration with spectroscopic OCT. In terms of PS contrast, this study focused only on phase retardation measurements which shows a clear definition of the RPE due to the high axial resolution. Additional future studies include the extension of PS-OCT to degree-of-polarization uniformity (DOPU) measurements in the retina, and axis orientation measurements in the RNFL specifically. The additional ability to perform a spectroscopic analysis of such polarization properties in the visible light range could provide an in vivo, non-invasive insight into the micro-structure of the murine retina.

## 5. Conclusion

A white light polarization sensitive optical coherence tomography system has been developed for sub-micron resolution imaging in the murine retina. Both healthy mice and VLDLR knockout mice have been imaged in vivo, demonstrating high resolution reflectivity images, phase retardation images and a spectroscopic contrast. A system such as this could prove a candidate for high resolution imaging in longitudinal studies of mouse models of retinal diseases.
